# Identification and *In-Silico* study of non-synonymous functional SNPs in the human *SCN9A* gene

**DOI:** 10.1371/journal.pone.0297367

**Published:** 2024-02-23

**Authors:** Sana Waheed, Kainat Ramzan, Sibtain Ahmad, Muhammad Saleem Khan, Muhammad Wajid, Hayat Ullah, Ali Umar, Rashid Iqbal, Riaz Ullah, Ahmed Bari

**Affiliations:** 1 Faculty of Life Science, Department of Zoology, University of Okara, Okara, Pakistan; 2 Faculty of Life Science, Department of Biochemistry, University of Okara, Okara, Pakistan; 3 Faculty of Animal Husbandry, Institute of Animal and Dairy Sciences, University of Agriculture, Faisalabad, Pakistan; 4 Department of Chemistry, University of Okara, Okara, Pakistan; 5 Faculty of Agriculture and Environment, Department of Agronomy, The Islamia University of Bahawalpur, Bahawalpur, Pakistan; 6 Department of Pharmacognosy College of Pharmacy King Saud University, Riyadh, Saudi Arabia; 7 Department of Pharmaceutical Chemistry, College of Pharmacy King Saud University, Riyadh, Saudi Arabia; Cukurova University Faculty of Medicine: Cukurova Universitesi Tip Fakultesi, TURKEY

## Abstract

Single nucleotide polymorphisms are the most common form of DNA alterations at the level of a single nucleotide in the genomic sequence. Genome-wide association studies (GWAS) were carried to identify potential risk genes or genomic regions by screening for SNPs associated with disease. Recent studies have shown that *SCN9A* comprises the NaV1.7 subunit, Na^+^ channels have a gene encoding of 1988 amino acids arranged into 4 domains, all with 6 transmembrane regions, and are mainly found in dorsal root ganglion (DRG) neurons and sympathetic ganglion neurons. Multiple forms of acute hypersensitivity conditions, such as primary erythermalgia, congenital analgesia, and paroxysmal pain syndrome have been linked to polymorphisms in the *SCN9A* gene. Under this study, we utilized a variety of computational tools to explore out nsSNPs that are potentially damaging to heath by modifying the structure or activity of the *SCN9A* protein. Over 14 potentially damaging and disease-causing nsSNPs (E1889D, L1802P, F1782V, D1778N, C1370Y, V1311M, Y1248H, F1237L, M936V, I929T, V877E, D743Y, C710W, D623H) were identified by a variety of algorithms, including SNPnexus, SNAP-2, PANTHER, PhD-SNP, SNP & GO, I-Mutant, and ConSurf. Homology modeling, structure validation, and protein-ligand interactions also were performed to confirm 5 notable substitutions (L1802P, F1782V, D1778N, V1311M, and M936V). Such nsSNPs may become the center of further studies into a variety of disorders brought by *SCN9A* dysfunction. Using *in-silico* strategies for assessing *SCN9A* genetic variations will aid in organizing large-scale investigations and developing targeted therapeutics for disorders linked to these variations.

## 1. Introduction

Single variant polymorphisms (SNVs) are segments of DNA with variations of only one base pair across individuals. The human genome has millions of SNPs that serve as biological markers due to the fact that individuals usually differ in one nucleotide out of every 1,000 or so [1–3]. Over half a million SNPs in the DNA coding sequence have been correlated to the emergence of previously unknown disorders [4,5]. Knowing which SNPs had an impact on the morphology was crucial for understanding the genetic basis of disease and phenotypic diversity, as well as deciding which markers to exploit in population-based association studies [6,7]. Missense mutations (nsSNPs) have a significant probability of inducing phenotypic variation in humans by modifying protein expression [8,9]. Many studies have found that nsSNPs account for about half of the DNA variations correlated with hereditary disorders [10]. GWAS studies explore those SNPs which are more common in people with the disease in order to identify genes which may contribute to disease susceptibility [11].

Further, *SCN9A* gene polymorphisms have been linked to a spectrum of pain perception, from extreme insensitivity to intense hypersensitivity, by encoding the alpha monomer of NaV1.7 channels was attributed to variations in pain sensitivity such as primary erythermalgia, congenital analgesia, and paroxysmal pain disorder [12–15]. Recent research have led to the discovery of variants that contribute to persistent pain disorders [15]. It is evident that abnormal transcription of voltage-gated Na^+^ channels (VGSCs) is a key mechanism causing a variety of diseases, notably migraine, pain, MS, and epilepsy [16]. Over the past decade, cellular-level research has provided insight about transmembrane channels and involved in the body pain response, such as the capsicum-activated TRPV1 heat receptor [17]. New studies have shown the catechol-O-methyltransferase (COMT) and tetrahydrobiopterinm role in pain perception that regulates nociceptors and chronic inflammation [18–20]. The NaV1.7 channels, often termed as the VGSCs-IV type subunit are made up of active pore-forming αlpha-monomers and accessory βeta-subunits and are needed for the formation of electrical impulses in nerve and endothelial cells, respectively [21–24]. In humans, nine distinct Na^+^ channels have been identified, each of which is encoded by a single *SCN1A-SCN9A* gene [18,25–28].

The *SCN9A* (NC 000002.12) gene encodes the Nav1.7 and spans 113.5 kb with 26 exons and mapped on human chromosome 2q24.3. Sodium channels produced by this gene and are mainly found in neurons of the dorsal root ganglion (DRG) and the sympathetic ganglia. Each domain of this Na^+^ channel, which contains 1988 amino acid, is split into six membrane segments [18–20,29–31]. A VGSC requires at least three subunits: an alpha-subunit and one or two βeta-subunits, which are much smaller. Each of the repetitive domains was tightly packed into the centre of the polypeptide chains, and the P-loop region between the S5 and S6 segment is essential for pore formation. There are four homology domains (DI-IV) in the alpha-subunit, and each of them splits the into six membrane segments (S1-6) [32]. Over evolutionary period, 9 distinct variants of the genes that generate the alpha monomer have emerged. Such genes have a broad array of tissue specificity and structural characteristics. Several diseases were linked to variations in a certain amino acid that occurred as a result of these gene alterations. These instances are tremor, dystonia, pain perception disorders, epilepsy, paralysis, ataxia, arrhythmia, cardiac and skeletal muscles disorder, and so more. They may also be associated with specific psychological problems. Researchers have discovered that *SCN9A* genetic mutation can cause a diverse variety of pain sensitivity, from extreme insensitivity to extreme sensitivity [20,29,33–35]. In this paper, we considered various *In-silico* approaches for pinpointing the human *SCN9A* non-synonymous polymorphisms.

## 2. Material and methods

**Fig 1:**
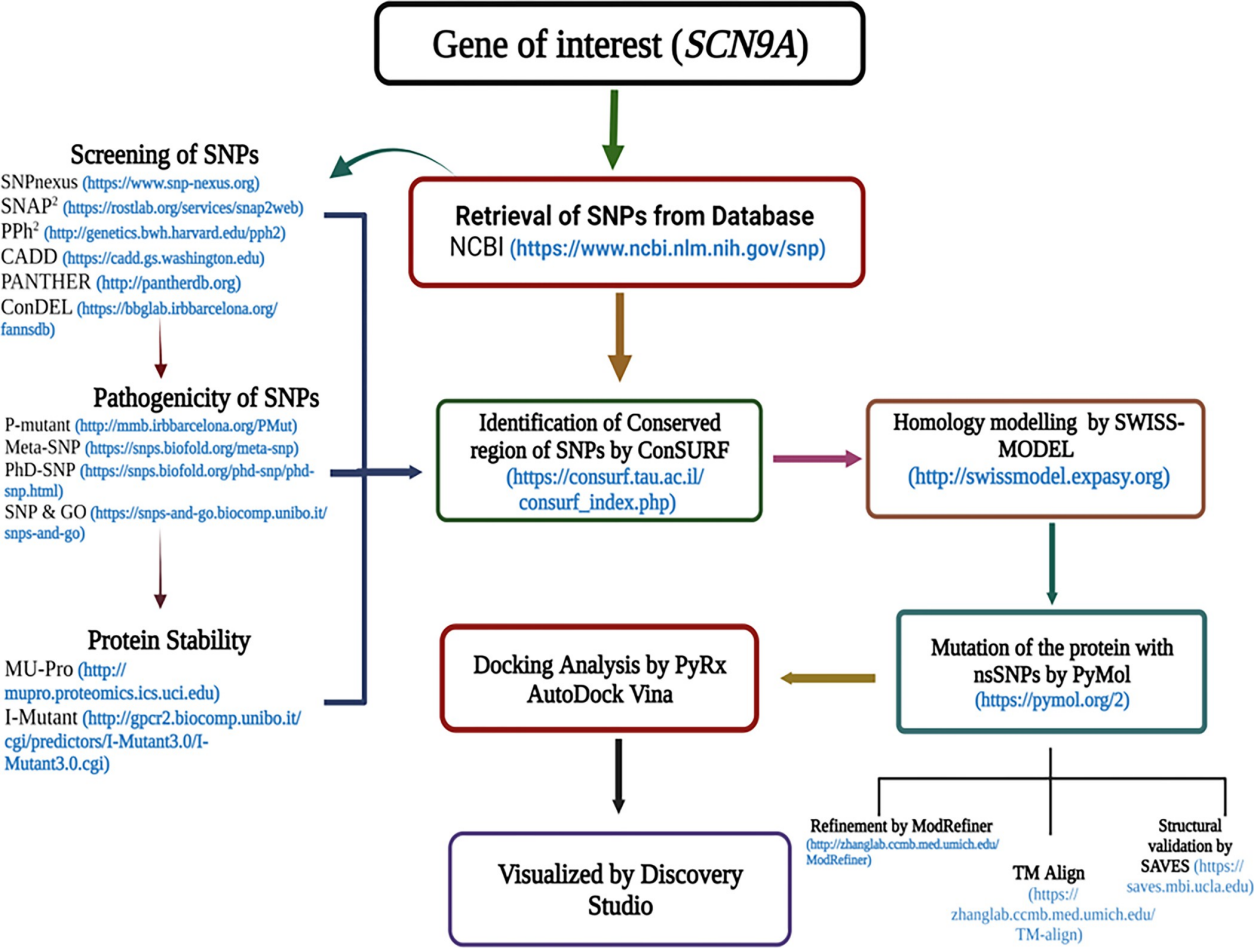
Entire workflow for nsSNPs screening in the *SCN9A* gene using computational tools.

The research methodology that was followed in this study is depicted schematically in **Fig 1.**

### 2.1. Retrieval of SNPs

Human *SCN9A* dbSNPs data was collected from a number of open-access databases, notably NCBI (http://www.ncbi.nlm.nih.gov) and the sequence was obtained from Uniprot (https://www.uniprot.org). Studies explored the detrimental impacts of missense SNPs on the *SCN9A* gene.

### 2.2. Assessment of deleterious consequences of SNPs

To assess the probable consequence of genetic variants retrieved from the dbSNP databases, we used a total of seven distinct bioinformatics tools. The aforementioned methods used: SNPnexus (https://www.snp-nexus.org) includes Sorting Intolerant from Tolerant (SIFT) and Polymorphism Phenotyping (PolyPhen) [36]. Based on the tolerance score, SIFT with a score of ≤0.05 is regarded as damaging, whereas with a value larger than the threshold is considered tolerant [36–39]. For each amino variant, Polyphen generates a position-specific independent count (PSIC) index. Differential PSIC scores for variations reveal the direct functional impact of polymorphisms on protein function [36,40–43]. SNAP2 (https://rostlab.org/services/snap2web) assess the effect (+100) or neutral (-100) of variants on the protein structures [44]. PPh-2 (http://genetics.bwh.harvard.edu/pph2) predicts how point mutations would affect protein expression [45]. It classifies mutations as possibly lethal (>0.15), probably damaging (>0.85), or benign depending on the existence or absent of protein substitutions in the query sequence [37,38,41,45,46]. CADD (https://cadd.gs.washington.edu) integrates a wide range of data into a single quantitative score and ranks genetic variants in the human genome such as single-base variations (SNVs) and Insertion/Deletion (InDels) [42,45]. Mutpred2 (http://mutpred2.mutdb.org/index.html) used to examine the potential structural implications of nsSNPs arising from protein alterations combined with biological and molecular evidence [47–49]. PANTHER (http://pantherdb.org) repository of biological and evolutionary evidence on all protein-coding genes [50]. It is a comprehensive resource for classification of genes according to their evolutionary history, and their functions [51]. ConDEL (https://bbglab.irbbarcelona.org/fannsdb) was created to examine the standardized scores from different algorithms, with the outcomes denoted as 0.0 for Neutral and 1.0 for Deleterious [52].

### 2.3. Screening of disease- associated SNPs

To examine the association of screened nsSNPs with a disease, P-Mu, PhD-SNP, and SNPs &GO were performed. Any variant with a p-value of greater than 0.5 was classified as a disease-associated nsSNPs. P-MUT (http://mmb.irbbarcelona.org/PMut) allows users to access all single amino acid variants on human proteins. It predicts pathological characteristics linked with a mutation in people with an 80% accuracy [53]. PhD-SNP (https://snps.biofold.org/phd-snp/phd-snp.html) with an accuracy of 78% and a score range of 0–9, this method may determine whether a mutation occurs is a benign polymorphic or is related to inherited mutations in humans [36,38,40,46,54]. SNP &GO (https://snps-and-go.biocomp.unibo.it/snps-and-go) assesses amino acid changes at a given locus in a particular protein [36–38,46]. Meta-SNP (https://snps.biofold.org/meta-snp) predicts disease when there are more than 0.5 mutations. Single predictor outputs are used as input in Meta-SNP, which achieves an overall accuracy of 79 percent [55].

### 2.4. Functional effects of SNPs on protein stability

To check the stability, I-Mutant (http://gpcr2.biocomp.unibo.it/cgi/predictors/I-Mutant3.0/I-Mutant3.0.cgi), a type of vector support machine-based web server that forecasts any modifications to protein stability upon being mutated [46]. As input, the *SCN9A* sequence and variants, temperature (25°C) and pH (7) were submitted. It predicts the reliability index of the outcomes on a range from zero to ten, where ten signifying the highest reliability [36,38,56]. MU Pro (http://mupro.proteomics.ics.uci.edu) evaluates protein sequence variations using wild and mutant residues, and a number below 0 (mutation has an effect on protein function), whereas a value larger than 0 indicates that the modification enhances protein stability [36,57].

### 2.5. Phylogenetic conservation analysis of nsSNPs

Using gene sequence, we calculated the conservation of amino acid sites through evolution by using ConSurf (https://consurf.tau.ac.il/consurf_index.php) [36,38,58]. The Bayesian approach resulted in a cutoff of conserved scores: Grading 1–4 as dynamic, 5–6 as moderate, and 7–9 as consistent [59,60]. Conserved patterns were anticipated from an input *SCN9A* FASTA sequence, yielding a conservation score and color scheme. For further study, we selected high-risk nsSNPs found in the highly conserved region [43].

### 2.6. Homology Modeling of *SCN9A* gene

Using the SWISS-MODEL platform (http://swissmodel.expasy.org), we built the 3D configurations of both wild-type and mutated proteins to predict structural stability and variations [61]. By homology modeling methods, the native *SCN9A* structure was modeled, and then subjected to a single point mutation in the pymol (https://pymol.org/2) [62]. The structure refinement was done by using ModRefiner (http://zhanglab.ccmb.med.umich.edu/ModRefiner) [63], and five separate refined protein models are provided at the end and each model will be downloaded as a PDB file [64].

### 2.7. Structural validation and RMSD calculation

The structural model was selected and subjected for the structural validation using SAVES server (https://saves.mbi.ucla.edu) The SAVES incorporates PROCHECK, and ERRAT to verify the quality of the whole 3D model. [41,65]. The model’s quality was also evaluated by RAMACHANDRAN plot generated by ProCHECK [36]. The 3D verification evaluates the concordance between a tertiary protein structure and its primary structure [66]. Afterwards TM-align (https://zhanglab.ccmb.med.umich.edu/TM-align) was used to compare wild type protein structure with mutant protein structures. In this method, we use a superposition to calculate both the template modeling score (TM-score) and the root mean square deviation (RMSD). TM-score provides a numeric value between 0 and 1, where 1 indicates a precise match between the two structures. A greater RMSD value is indicative of greater structural divergence between wild-type and mutant forms [67–69]. The RAMAHANDRAN Plot also considered the dihedral angle of atoms in amino acid residues to pinpoint the preferred region of amino acids [70–72].

### 2.8. Protein–Ligand docking analysis

Molecular docking was performed in order to find ligand protein interaction and for finding potential ligands. For this, we docked all selected ligands with SCN9A using PyRx program (https://pyrx.sourceforge.io). The Lamarckian genetic algorithm (LGA) which incorporates AutoDock and AutoDock Vina, was applied for virtual ligand screening [73–75]. The 10 greatest exclusive values were calculated for each ligand, with the active parameters set to the grid size of the center (XYZ axis). The AutoDock tools were used to convert the PDB files to Pdbqt format and calculate the binding affinities [76]. For virtual screening, Discovery Studio (https://discover.3ds.com/discovery-studio-visualizer-download) was used for 2D and 3D interaction of ligands with protein. They showed the size and location of bonding sites, hydrogen bond interactions, hydrophobic interactions, and bonding distances of a docked ligand [77,78].

## 3. Results

### 3.1. Download SNPs datasets

**Fig 2:**
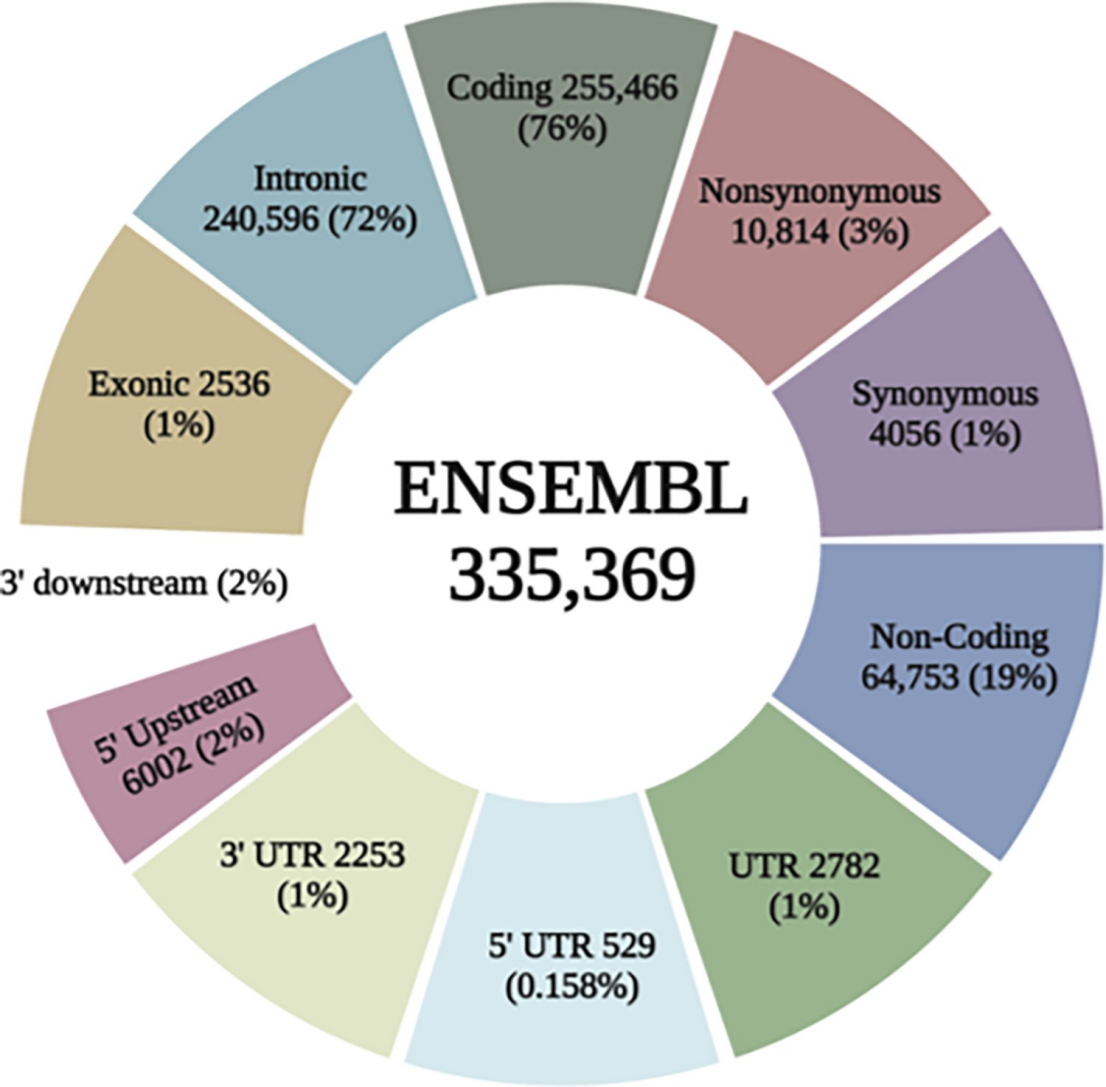
Pie chart distribution of Single nucleotide polymorphisms (SNPs) in *SCN9A* gene.

According to the NCBI dbSNP database, the human *SCN9A* gene had 335369 SNPs. Exons are DNA fragments that undergo translation after introns are eliminated during splicing, and is often utilized to refer a protein-coding regions, which is erroneous, particularly in humans, where less than 30% of exonic sequences code for proteins. It is critical to comprehend that exons and introns may also be found in untranslated regions (UTRs), both in the 5’ and 3’ UTRs. The study of SNPs in such UTRs sheds light on the functional and structural repercussions of uncommon exonic SNPs in the human genome. The human *SCN9A* gene comprises of 26 coding exons, and the 3’ UTRs of *SCN9A* are highly similar with around 80% sequence similarity between the human and mouse genes. It highlights the significance of controlling *SCN9A* gene across species. From Ensembl (335369), 2,782 SNPs found in UTRs, synonymous (4,056), non-synonymous (10418), exonic (2536), intronic (240596), 3’UTR (2243), 5’UTR (529), non-coding (64753), and 255,466 SNPs became identified within coding region as shown in **Fig 2**. The *SCN9A* SNPs were assessed further in this study to anticipate their impact on target protein, stability, and activity and only nsSNPs were evaluated for further investigation.

### 3.2. Functional prediction of deleterious SNPs

**Fig 3:**
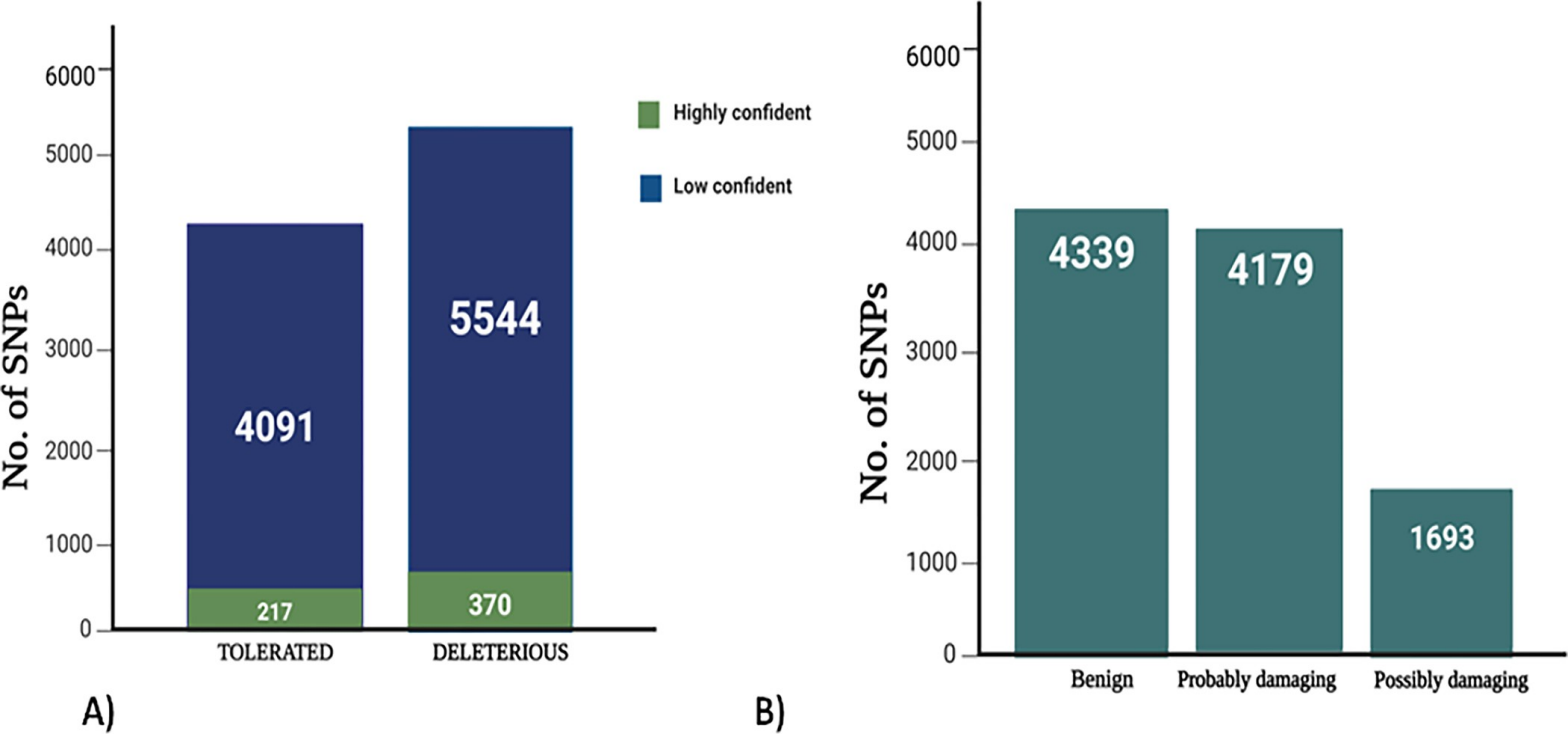
Prediction of functional consequences of nsSNPs by A) SIFT and B) PolyPhen.

The retrieved 66326 missense SNPs were analyzed first using SNPnexus and assigned each one an index value. In the SIFT algorithm, 5914 nsSNPs are found as deleterious out of 5544 missense SNPs that may have a functional effect on the protein. The output value for Polyphen varies from 0 to 1, with 1 being the most damaging and 0 shows neutral behavior. Among nsSNPs, 1693 nsSNPs are possibly damaging and 4339 were benign and 4179 nsSNPs are highly deleterious as shown in **Fig 3.** We selected common nsSNPs that scored 0 in SIFT and 1 in PolyPhen to ensure that only the most detrimental SNPs would be studied. We found 18 nsSNPs out of 132 that met the criterion and categorized them as high risk of affecting protein function. The complete data of commonly found 18 nsSNPs are given in **Table 1.** With a range from -100 to +100, SNAP-2 evaluates the no influence of one amino acid residue. SNAP-2 indicated that 3 nsSNPs including C710W (rs1358344300), D743Y (rs1186212683) and C1370Y (rs1390718765) shows a highest score of 90, 89 and 86 as shown in **Table 1.**

**Table 1:** High risk non-synonymous SNPs identified by SIFT, Polyphen, Mutpred2 and SNAP2.

Variant ID	Nucleotides	AA Variations	SIFT Score	Prediction	Polyphen	Prediction	SNAP2 Effect	Score	MutPred2
					Score				Score
rs201035087	C/G	E1889D	0	Deleterious	1	Probably Damaging	Effect	69	0.636
rs1277590831	A/G	L1802P	0	Deleterious	1	Probably Damaging	Effect	71	0.941
rs767347325	A/C	F1782V	0	Deleterious	1	Probably Damaging	Effect	46	0.805
rs760665758	C/T	D1778N	0	Deleterious	1	Probably Damaging	Effect	67	0.608
rs376892319	G/A	P1706S	0	Deleterious	1	Probably Damaging	Effect	31	0.777
rs1390718765	C/T	C1370Y	0	Deleterious	1	Probably Damaging	Effect	86	0.948
rs752362481	C/T	V1311M	0	Deleterious	1	Probably Damaging	Effect	41	0.887
rs1173515969	T/C	T1291A	0	Deleterious	1	Probably Damaging	Effect	50	0.857
rs1223854779	A/G	Y1248H	0	Deleterious	1	Probably Damaging	Effect	58	0.808
rs1269325856	G/T	F1237L	0	Deleterious	1	Probably Damaging	Effect	57	0.902
rs1451456893	T/C	M936V	0	Deleterious	1	Probably Damaging	Effect	39	0.944
rs757870879	A/G	I929T	0	Deleterious	1	Probably Damaging	Effect	51	0.936
rs529022680	C/A	V877F	0	Deleterious	1	Probably Damaging	Effect	82	0.911
rs1186212683	C/A	D743Y	0	Deleterious	1	Probably Damaging	Effect	89	0.943
rs1010654142	C/A	W730L	0	Deleterious	1	Probably Damaging	Effect	63	0.865
rs1358344300	A/C	C710W	0	Deleterious	1	Probably Damaging	Effect	90	0.912
rs200398202	C/G	D623H	0	Deleterious	1	Probably Damaging	Effect	71	0.79
rs947776327	C/T	M358I	0	Deleterious	1	Probably Damaging	Effect	2	**0.873**

In PPh-2, 18 nsSNPs were projected to be detrimental (PSIC > 0.5); 18 of these variants were anticipated to be highly damaging, with a PSIC score of 1. MutPred2 to assess the 18 prevalent SNPs for their possible effects and were expected to be deleterious to the *SCN9A* protein based on the results, with scores ranging from 0.636 to 0.948 and P values less than 0.05. Scores with P < 0.05 and g > 0.5 indicated an actionable hypothesis, scores with P < 0.05 and g > 0.75 indicated a confident hypothesis, and scores with P < 0.01 and g > 0.75 were classed as very confident hypotheses based on the mechanistic disruption induced by the nsSNPs mutations. As a result, all of the selected nsSNPs were categorized as very confident or highly probable to be detrimental hypotheses. The PANTHER tool predicts whether the nsSNPs will influence the protein function. For 18 nsSNPs, the estimated score was equal to or less than 3, leading in a probability of adverse effect greater than 0.5. From CADD values range from non-deleterious to deleterious, with greater scores indicates the highly damaging polymorphism. In addition, ConDEL prediction shows 18 nsSNPs have deleterious effect on *SCN9A* gene and **Table 2** shows the complete forecast outcomes.

**Table 2:** Cumulative prediction of possible deleterious nature of nsSNPs.

Variant ID	Nucleotides	AA Variations	PPh-2 Score	Prediction	PANTHER	CADD Score	ConDEL Score	Prediction
rs201035087	C/G	E1889D	1	Probably Damaging	Probably Damaging	23.4	0.6604	Deleterious
rs1277590831	A/G	L1802P	1	Probably Damaging	Probably Damaging	25.1	0.55786	Deleterious
rs767347325	A/C	F1782V	1	Probably Damaging	Probably Damaging	28.3	0.52343	Deleterious
rs760665758	C/T	D1778N	1	Probably Damaging	Probably Damaging	26.7	0.53728	Deleterious
rs376892319	G/A	P1706S	1	Probably Damaging	Probably Damaging	26.4	0.6071	Deleterious
rs1390718765	C/T	C1370Y	1	Probably Damaging	Probably Damaging	26.3	0.74338	Deleterious
rs752362481	C/T	V1311M	1	Probably Damaging	Probably Damaging	25.4	0.68273	Deleterious
rs1173515969	T/C	T1291A	1	Probably Damaging	Probably Damaging	28.6	0.585421	Deleterious
rs1223854779	A/G	Y1248H	1	Probably Damaging	Probably Damaging	28.9	0.645473	Deleterious
rs1269325856	G/T	F1237L	1	Probably Damaging	Probably Damaging	24.9	0.732668	Deleterious
rs1451456893	T/C	M936V	1	Probably Damaging	Probably Damaging	26.2	0.571746	Deleterious
rs757870879	A/G	I929T	1	Probably Damaging	Probably Damaging	28.2	0.573044	Deleterious
rs529022680	C/A	V877F	1	Probably Damaging	Probably Damaging	26	0.535492	Deleterious
rs1186212683	C/A	D743Y	1	Probably Damaging	Probably Damaging	28.7	0.714423	Deleterious
rs1010654142	C/A	W730L	1	Probably Damaging	Probably Damaging	42	0.713647	Deleterious
rs1358344300	A/C	C710W	1	Probably Damaging	Probably Damaging	38	0.695742	Deleterious
rs200398202	C/G	D623H	1	Probably Damaging	Probably Damaging	26.4	0.656425	Deleterious
rs947776327	C/T	M358I	1	Probably Damaging	Probably Damaging	26.8	0.58972	Deleterious

*PPh2—Polyphen2.

### 3.3. Prediction of disease causing nsSNPs

The SNP & GO algorithm identified 17 nsSNPs that were related with disease, while the mutation T1291A (rs1173515969) was graded as neutral. The prediction results are summarized in **Table 3.** P-Mutant indicates a certain 15nsSNPs will cause disease, which classify them as TRUE, While P1706S, T1291A and V877F predict FALSE result. As shown in **Table 3**, Meta-SNP predicts 2 nsSNPs (P1706S, T1291A) shows neutral effect on SCN9A protein. The prediction score (<0.5) makes the SNP neutral and disease causing (>0.5). PhD-SNP found only 5 nsSNPs were neutral including E1889D, P1706S, T1291A, I929T and M358I and remaining were disease causing, as shown in the **Table 3**.

**Table 3:** Prediction of disease causing SNPs by P-Mutant, Meta SNP, PhD SNP and SNP & GO.

Variant ID	Nucleotides	AA Variations	P-Mutant		Meta SNP		PhD SNP		SNP & GO	
rs201035087	C/G	E1889D	0.5263	TRUE	Disease	1	Neutral	0.477	Disease	1
rs1277590831	A/G	L1802P	0.6997	TRUE	Disease	7	Disease	0.853	Disease	3
rs767347325	A/C	F1782V	0.882	TRUE	Disease	4	Disease	0.828	Disease	3
rs760665758	C/T	D1778N	0.8536	TRUE	Disease	3	Disease	0.566	Disease	2
rs376892319	G/A	P1706S	0.3946	FALSE	Neutral	0	Neutral	0.163	Disease	1
rs1390718765	C/T	C1370Y	0.8652	TRUE	Disease	4	Disease	0.893	Disease	4
rs752362481	C/T	V1311M	0.8184	TRUE	Disease	1	Disease	0.731	Disease	4
rs1173515969	T/C	T1291A	0.4511	FALSE	Neutral	5	Neutral	0.159	Neutral	8
rs1223854779	A/G	Y1248H	0.7505	TRUE	Disease	5	Disease	0.728	Disease	4
rs1269325856	G/T	F1237L	0.8398	TRUE	Disease	6	Disease	0.917	Disease	3
rs1451456893	T/C	M936V	0.8577	TRUE	Disease	3	Disease	0.583	Disease	7
rs757870879	A/G	I929T	0.7922	TRUE	Disease	3	Neutral	0.29	Disease	7
rs529022680	C/A	V877F	0.4994	FALSE	Disease	4	Disease	0.561	Disease	8
rs1186212683	C/A	D743Y	0.8492	TRUE	Disease	6	Disease	0.798	Disease	8
rs1010654142	C/A	W730L	0.882	TRUE	Disease	8	Disease	0.877	Disease	8
rs1358344300	A/C	C710W	0.7855	TRUE	Disease	7	Disease	0.836	Disease	9
rs200398202	C/G	D623H	0.6523	TRUE	Disease	4	Disease	0.76	Disease	7
rs947776327	C/T	M358I	0.5567	TRUE	Disease	2	Neutral	0.287	Disease	4

### 3.4. Prediction of *SCN9A* protein stability

The DDG forecasted by I-Mutant 3.0 identified 5 mutations were expected to increase the stability of the mutant protein, whereas the remaining 13 nsSNPs were predicted to decrease the stability of the protein, hence lowering protein activity. Disease-related nsSNPs were predicted using a sequence-based method from the I-Mutant 3.0 package. Findings for the estimated structural influence of 18 potential nsSNPs were obtained from Mu-Pro servers. The findings of the protein stability assessment are listed in **Table 4**.

**Table 4:** List of nsSNP’s predicted by Mu-Pro, I mutant and ConSURF.

Variant ID	Nucleotides	AA Variations	MU Pro	Score	I mutant	Score	ConSurf
rs201035087	C/G	E1889D	Decrease	-1.003035	Decrease	5	9,e,f
rs1277590831	A/G	L1802P	Decrease	-2.0284824	Decrease	2	9,b,s
rs767347325	A/C	F1782V	Decrease	-0.5691432	Decrease	8	9,b,s
rs760665758	C/T	D1778N	Decrease	-0.9162488	Decrease	5	9,e,f
rs376892319	G/A	P1706S	Decrease	-1.753187	Decrease	8	9,b,s
rs1390718765	C/T	C1370Y	Decrease	-1.1410874	-	-	9,b,s
rs752362481	C/T	V1311M	Decrease	-0.3113898	Decrease	6	9,b,s
rs1173515969	T/C	T1291A	Decrease	-0.6659933	Decrease	8	9,b,s
rs1223854779	A/G	Y1248H	Decrease	-1.5449369	Decrease	9	8,e,f
rs1269325856	G/T	F1237L	Decrease	-1.1002138	Increase	5	9,b,s
rs1451456893	T/C	M936V	Decrease	-0.5491587	Decrease	8	9,b,s
rs757870879	A/G	I929T	Decrease	-1.0086574	Decrease	7	9,b,s
rs529022680	C/A	V877F	Decrease	-1.162129	Decrease	8	9,b,s
rs1186212683	C/A	D743Y	Decrease	-0.828799	Increase	1	9,b,s
rs1010654142	C/A	W730L	Decrease	-0.330446	Increase	4	8,b
rs1358344300	A/C	C710W	Decrease	-0.8210315	Decrease	4	8,b
rs200398202	C/G	D623H	Decrease	-1.0159569	Decrease	7	7,e
rs947776327	C/T	M358I	Decrease	-0.6336562	Increase	3	9,b,s

### 3.5. Evolutionary conservation of deleterious nsSNPs

**Fig 4:**
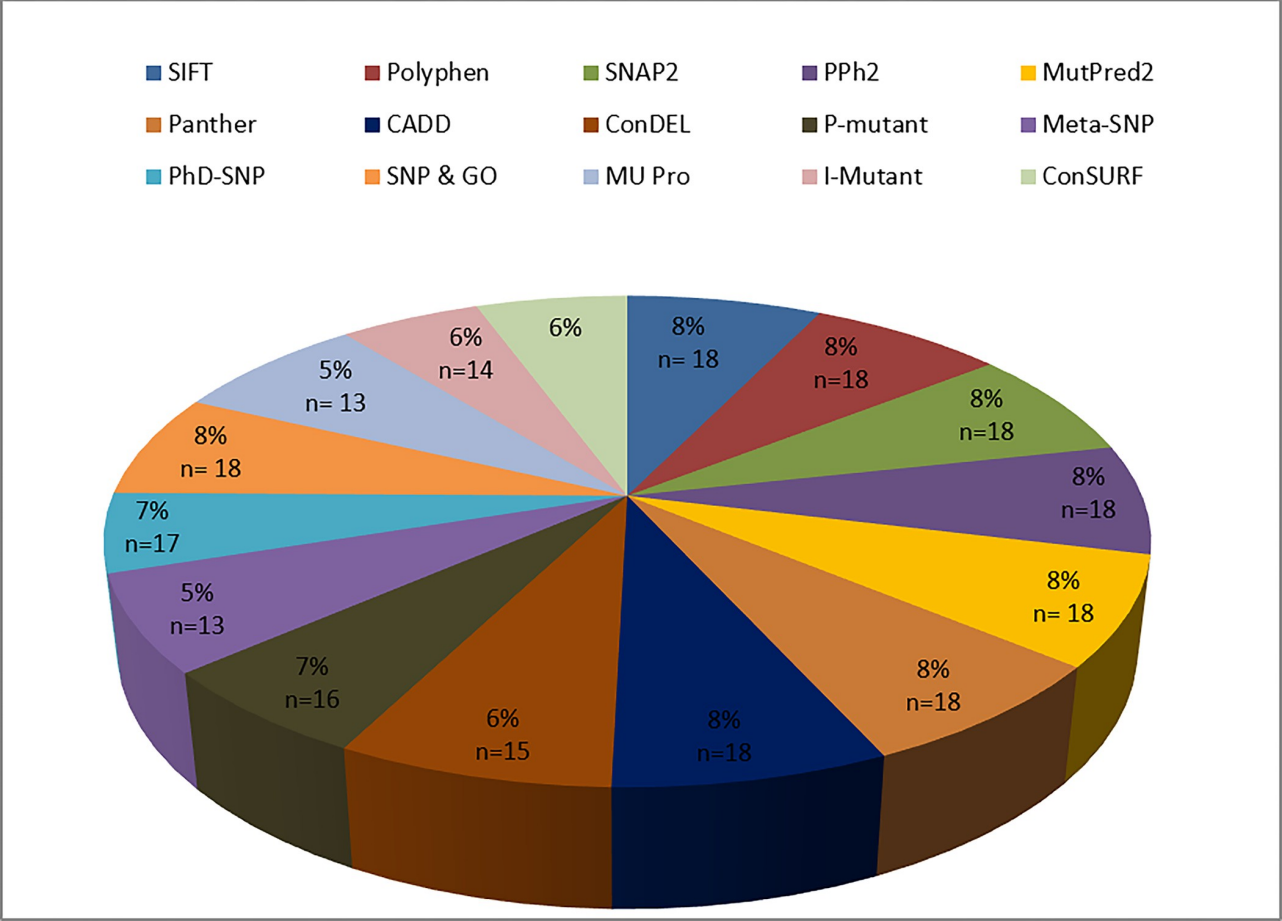
Pie chart displaying the prevalence of deleterious missense mutations. Evaluation of 15 *In silico* tools reveals the percentage and numerical quantity of deleterious nsSNPs.

The ConSurf analysis revealed a high degree of structural and functional conservation among all *SCN9A* residues. On the other hand, we focused on solely on the 9-scoring residues that corresponded to the 14 high-risk nsSNPs we discovered. According to the findings, Y1248H are highly exposed as they are functional residues. It can be shown in **Table 4** that C710W (rs1358344300) and W730L (rs1010654142) are projected to be structural residues, which indicates that they are deeply buried. **Fig 4** displays the data that proved these 14 high-risk nsSNPs to be truly detrimental to the structure and/or function of the *SCN9A* protein.

### 3.6. SWISS modeling for the *SCN9A* protein

**Fig 5:**
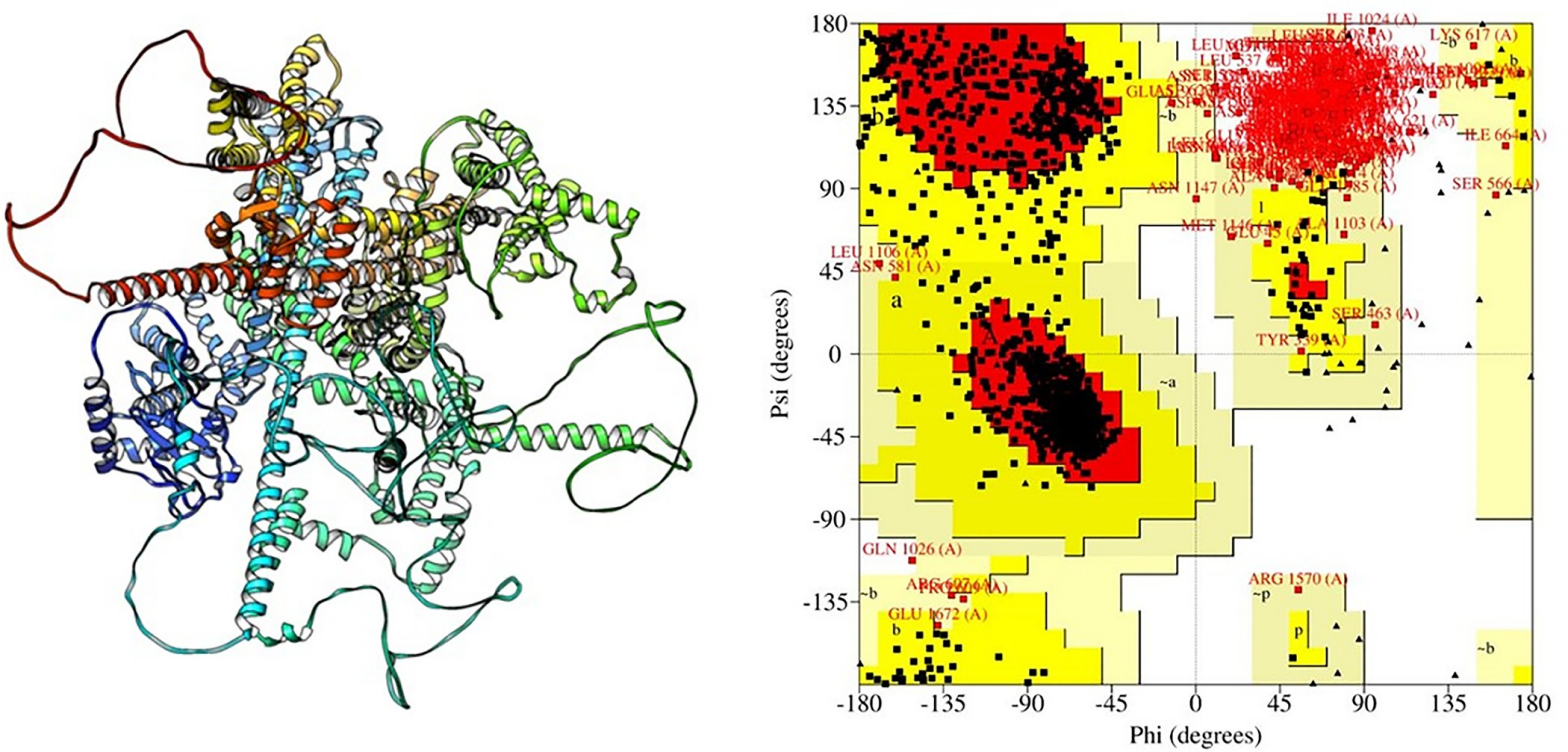
Procheck-RAMACHANDRAN plot of the native *SCN9A* predicted model.

The prediction score indicates that 14 highly-conserved mutations on the *SCN9A* protein were evaluated to detect the protein conformational modifications induced by them. The list of those 14 nsSNPs are as follows; E1889D, L1802P, F1782V, D1778N, C1370Y, V1311M, Y1248H, F1237L, M936V, I929T, V877E, D743Y, C710W, D623H. For comparative homology modeling the generated sequences were selected of at least >30% similarities and identities. We got 50 templates with 64.41% STML ID 6Iqa.1.A identification for the query sequence. To learn how mutations drastically alter the stability of proteins, we modeled the three-dimensional structure of the *SCN9A* protein using the template PDB ID 6lqa.1. A (range: 2-1978aa). The results obtained by using a template with a quality of 6lqa.1 and then using PyMOL to create a model are displayed in **Fig 5.**

The most favored, additional allowed, generously allowed, and disallowed regions are colored in red, yellow, light yellow, and white respectively.

The above listed proteins have been downloaded with the respective PDB files and mutated by PyMol. The mutants (L1802P, F1782V, D1778N, V1311M, and M936V) showing high RMSD value as shown in **Table 5**. Validation of the modeled framework was performed by SAVES and RAMACHANDRAN plot evaluation was used to examine at the secondary structure. All constraints imposed by potential energy calculations were respected by the resulting structure. On a RAMACHANDRAN plot, the majority of the amino acid residues in the *SCN9A* protein (82.90%) were found in a highly favorable region as depicted in **Fig 5**. The complete predicted results can be found in **Table 5.**

**Table 5:** Structural validation and comparison of *SCN9A* gene.

	ERRAT	PROCHECK				TM Align	
Variation ID	Score	Core	Allow	Generously	Disallowed	RMSD	TM score
E1889D	86.569	82.80%	12.00%	2.80%	2.40%	0.25	0.99787
L1802P	84.3731	82.10%	11.60%	3.00%	3.20%	0.36	0.99757
F1782V	83.7886	82.10%	11.80%	3.00%	3.10%	0.33	0.99833
D1778N	88.5607	81.60%	12.60%	3.30%	2.50%	0.35	0.99759
C1370Y	85.8092	81.80%	12.60%	3.20%	2.40%	0.31	0.99815
V1311M	85.7669	81.90%	11.60%	3.70%	2.80%	0.35	0.99799
Y1248H	85.0478	82.30%	12.20%	2.80%	2.70%	0.34	0.99779
F1237L	86.724	82.50%	12.50%	2.70%	2.30%	0.29	0.99745
M936V	83.5866	82.20%	11.60%	3.70%	2.50%	0.36	0.99738
I929T	86.4583	82.80%	12.00%	2.90%	2.40%	0.25	0.9977
V877E	84.8745	82.70%	11.40%	3.30%	2.70%	0.25	0.99761
D743Y	85.954	82.90%	11.60%	3.50%	2.00%	0.34	0.99781
C710W	84.297	82.00%	12.50%	3.20%	2.30%	0.32	0.99763
D623H	84.6386	82.70%	12.20%	2.90%	2.20%	0.31	0.99731

### 3.7. Molecular Docking by PyRx

To discover ligand-protein interactions, molecular docking was used by PyRx tool; we docked all of the selected ligands with *SCN9A*. They created ten distinct conformations for each ligand, which are characterized by binding affinity (-Kcal/mol).The docking results of ligands indicates that these binding affinities relate to their level of activity, and all 20 compounds with binding affinities are given in **Table 6.** We selected 9 compounds with strong binding affinities, notably batrachotoxin, carbamazepine, flecainide, funapide, naloxone, phenytoin, ranolazine, saxitoxin, and vixotrigine, and docked them with all 6 of our native and mutated protein complexes further to investigate their interactions. Discovery studio, which offers a 2D representation of all docking interactions, was used for the research.

**Table 6:** Top ranked binding affinities of 20 compounds with native and mutant proteins.

Ligands	SCN9A Wild	L1802P	F1782V	D1778N	V1311M	M936V
Batrachotoxin	-7.7	-7.5	-7.5	-7.6	-7.5	-7.5
Benzazepinone	-6.1	-6.2	-6.2	-6.2	-6.2	-6.2
Carbamazepine	-6.7	-6.9	-6.9	-7.8	-6.9	-6.9
Chromane	-5.2	-5.4	-5.4	-5.4	-5.4	-5.4
Flecainide	-6.3	-6.8	-6.8	-6.8	-6.8	-7
Funapide	-7.5	-8.3	-8.2	-8.3	-8.2	-8.3
Ascorbic acid	-4.4	-5.1	-5.1	-5.1	-5.1	-5.1
Lacosamide	-5.4	-6.1	-6.1	-6.1	-6.1	-6.1
Lamotrigine	-5.8	-6	-5.7	-6	-5.9	-5.9
Lidocaine	-5.4	-6	-6	-6	-6	-5.9
Mexiletine	-5.1	-5.4	-5.4	-5.4	-5.4	-5.4
Naloxone	-6.9	-7.2	-7.3	-7.2	-6.2	-7.2
Phenytoin	-6.9	-7.7	-7.7	-6.7	-6.7	-7.7
Ralfinamide	-6.8	-6.3	-6.3	-6.3	-6.3	-6.3
Ranolazine	-6.6	-6.9	-6.8	-6.9	-6.7	-6.6
Riluzole	-5.9	-5.6	-5.6	-5.7	-5.6	-5.6
Saxitoxin	-6.9	-5.7	-5.7	-5.7	-5.8	-5.7
Tetrodotoxin	-6.3	-6	-5.9	-5.9	-6	-6
Topiramate	-5.8	-5.8	-5.8	-5.8	-5.8	-5.8
Vixotrigine	-6.8	-6.7	-6.8	-6.7	-6.7	-6.7

**Fig 6:**
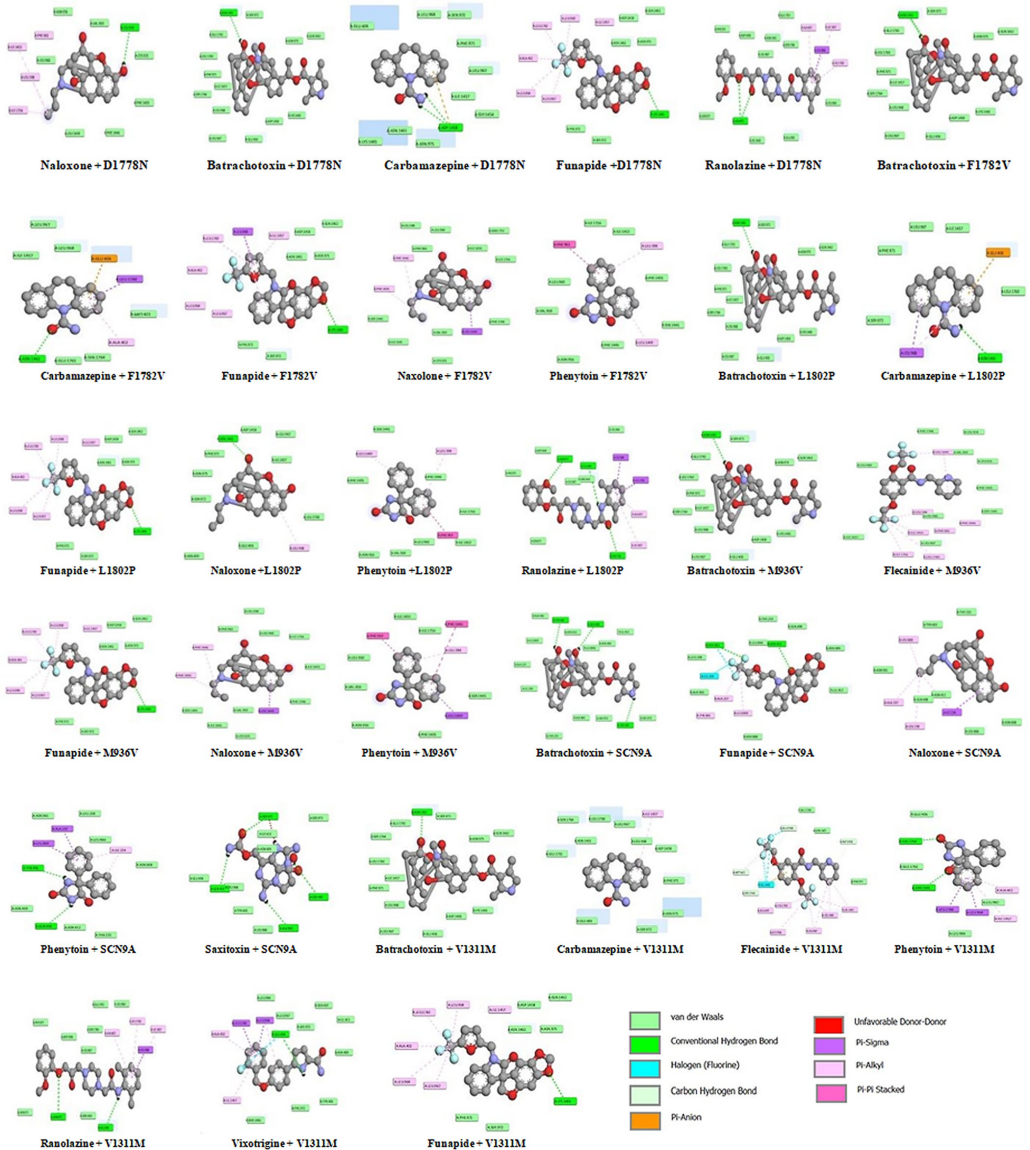
Interaction of protein ligands with typical *SCN9A* and mutant D1778N, F1782V, L1802P, M939V, and V1311M.

The binding free energy of all the chosen ligands is higher than -4Kcal/mol. The highest binding energy revealed that the *SCN9A* protein docked successfully with Batrachotoxin. The Batrachotoxin and Funapide shows highest binding affinity -7.7 and -7.5Kcal/mol, which are greater than other ligand-binding affinities. A Batrachotoxin ligand was fixed in the SCN9A binding pocket sites by forming the conventional hydrogen bond with residues TYR 405, GLN 408, and SER 969; and Vander wall interactions with ALA 965, ASN 412, LEU 866, ASN 409, ILE 413, SER 973, SER 972, ALA 865, THR 233, ILE 234, ALA 237 and LEU 869. The interacting residues obtained from docking are presented in **Fig 6.** Differences between mutant and natural ligand-protein residue interactions, indicative of altered functional features due to mutations, are listed in **Table 7**.

**Table 7:** Interacting residues obtained from docking. Wild protein, L1802P, F1782V, D1778N, V1311M and M936V variants with ligands including their binding residues and hydrophobic interactions.

Protein-Ligands	Hydrogen bond Interactions	Hydrophobic Interactions	Protein-Ligands	Hydrogen bond Interactions	Hydrophobic Interactions
Wild- Batrachotoxin	TYR 405, GLN408, SER 969, ALA965, LEU869, ALA237, ILE234, THR233, ALA865, SER972, SER873, LEU866, ASN409, ILE413, ASN412	NULL	L1802P- Batrachotoxin	SER972, ASN1461, ASN975, GLN1462, LYS1465, ASP1458, GLU406, LEU967, LEU968, SER1764, ILE1457, PHE871, LEU1760, GLU1761	NULL
Wild- Funapide	THR233, GLN408, ASN409, ILE413, LEU866, ASN868, ALA965, LEU238, ASN961, ASN412	ILE234, ALA237, LEU898, TYR405	L1802P- Carbamazepine	LEU967, ILE1457, ILE1760, SER972, PHE971	LEU968, GLU406
Wild- Naloxone	TYR405, THR233, LEU866, ASN868, ASN412, GLN408, ASN961	LEU869,ALA237, LEU238,ILE234	L1802P- Funapide	ASP1458, GLN1462, ASN1461, ASN975, PHE971, SER972, LYS1465	LEU968, LEU1760, LEU964, LEU967, ALA402, ILE1457
Wild- Phenytoin	ASN961, LEU238, LEU866, ASN868, ASN412, ASN409, TYR405, GLN408	ILE234, ALA237, LEU869,	L1802P- Naloxone	ASP1458, LEU967, ILE1457, LEU1760, GLU406, ASN409, SER972, ASN975, PHE971	LEU968, ASN1461
Wild- Saxitoxin	ILE413, SER973, LEU866, TYR405, LEU968, GLU406, GLN410, ALA965, SER969, SER972	NULL	L1802P- Phenytoin	SER1445, PHE1446, ILE1756, ILE1453, LEU960, VAL959, ASN956, PHE1405	PHE963, LEU1449, LEU398
F1782V- Batrachotoxin	SER972, ASN1461, ASN975, GLN1462, LYS1465, ASP1458, GLU406, LEU967, LEU968, SER1764, ILE1457, PHE871, LEU1760, GLU1761	NULL	L1802P- Ranolazine	ASN975, GLU406, SER1764, ASP1458, LEU967, ASN1461, LEU964, ASN1461, LEY964, SER972, PHE971	LEU968, LEU1760, ALA402, ILE1457
F1782V- Carbamazepine	GLU1761, SER1764, MET403, LEU957, LEU968, ILE1457	GLU406, LEU1760, ALA402	V1311M- Batrachotoxin	SER972, ASN1461, ASN975, GLN1462, LYS1465, ASP1458, GLU406, LEU967, LEU968, SER1764, ILE1457, PHE871, LEU1760, GLU1761	NULL
F1782V-Funapide	ASP1458, GLN1462, ASN1461, ASN975, PHE971, SER972, LYS1465	LEU968, LEU1760, LEU964, LEU967, ALA402, ILE1457	V1311M- Carbamazepine	SER1764, LEU1760, LEU967, LEU968, ASP1458, PHE971, ASN975, SER972, GLU406, ASN1461, GLU1761	ILE1457
F1782V- Naloxone	LEU398, LEU960, PHE963, ILE 1453, ASN 1753, ILE1756, PHE1748, VAL959, CYS925, ILE1441, SER1445	LEU1449, PHE1446, PHE1405	V1311M- Flecainide	ASP1458, MET403, SER1764, GLU1761, ASN1461, PHE971	GLU406, ILE1457, LEU968, LEU967, LEU1760, LEU964, ALA402
F1782V- Phenytoin	ILE1756, ILE1453, PHE1405, SER1445, PHE1446, ASN956, VAL959, LEU960	PHE963, LEU398, LEU1449	V1311M- Funapide	ASP1458, GLN1462, ASN1461, ASN975, PHE971, SER972, LYS1465	LEU968, LEU1760, LEU964, LEU967, ALA402, ILE1457
D1778N- Batrachotoxin	SER972, ASN1461, ASN975, GLN1462, LYS1465, ASP1458, GLU406, LEU967, LEU968, SER1764, ILE1457, PHE871, LEU1760, GLU1761	NULL	V1311M- Phenytoin	ASN1461, SER1764, GLU406, GLU1761, LEU964, LEU967	LEU1760, LEI968, ILE1457, ALA402
D1778N- Carbamazepine	ASP1458, GLU406, LEU968, SER972, PHE971, LEU967, ILE1457, GLY1454, ASN975, ASN1461, LYS1465	ASP1458	V1311M- Ranolazine	PHE971, ASP1458, ASN1461, LEU967, SER1764, GLU1761, GLU406,LYS1465, LEU964, SER772, ASN975	LEU968, ALA402, ILE1457
D1778N- Funapide	ASP1458, GLN1462, ASN1461, ASN975, PHE971, SER972, LYS1465	LEU968, LEU1760, LEU964, LEU967, ALA402, ILE1457	V1311M- Vixotrigine	LEU967, GLN410, LEU964, ILE413, SER972, ASN409, TYR405, PHE971, ASN1461	LEU1760, LEU968, ALA402, ILE1457, GLU406
D1778N-Naloxone	LEU924, ASN965, VAL959, PHE1405, PHE1446, LEU1449, LEU960, CYS925	PHE963, ILE1453, LEU398, ILE1756	M936V - Batrachotoxin	SER972, ASN1461, ASN975, GLN1462, LYS1465, ASP1458, GLU406, LEU967, LEU968, SER1764, ILE1457, PHE871, LEU1760, GLU1761	NULL
D1778N-Ranolazine	PHE971, ASP1458, ASN1461, LEU967, SER1764, GLU1761, GLU406,LYS1465, LEU964, SER772, ASN975	LEU968, ALA402, ILE1457	M936V- Flecainide	PHE1748, LEU924, VAL1959, CYS925, PHE1405, SER1445, LEU964, LEU967, ILE1457, LEU960	LEU1449, PHE1446, LEU398,PHE963, ILE1453, ILE1756, LEU1760
M936V- Naloxone	LEU398, LEU960, PHE963, ILE 1453, ASN 1753, ILE1756, PHE1748, VAL959, CYS925, ILE1441, SER1445	LEU1449, PHE1446, PHE1405	M936V- Funapide	ASP1458, GLN1462, ASN1461, ASN975, PHE971, SER972, LYS1465	LEU968, LEU1760, LEU964, LEU967, ALA402, ILE1457
M936V- Phenytoin	ILE1756, ILE1453, PHE1405, SER1445, PHE1446, ASN956, VAL959, LEU960	PHE1446, PHE963, LEU1449			

According to Table 7, polymorphisms not only alter the protein’s conformation because of modifications to the number of interacting residues, but also because of alterations in hydrogen bonds and hydrophilic associations.

## 3. Discussion

The prevalence of deleterious non-synonymous substitutions in the *SCN9A* gene was determined by evaluating 15 different *In-Silico* analyses. This difference highlights the need for a comparative evaluation to accurately identify the nsSNPs that often significantly impair *SCN9A* gene activity. Consequently, we used a meta-analysis of 14 different computational methods to determine how to properly classify nsSNPs, from benign to detrimental. We focused on the 18 nsSNPs identified as deleterious by SNPnexus and that were classified as harmful by the 2 tools (Table 1). More than five algorithms showed high consistency in forecasting of the nsSNPs were harmful, deleterious, or disease-associated. Despite the fact that most of the 18 most damaging nsSNPs identified here were not tested in vitro, and there is no information on the functional significance of these mutations in *SCN9A* protein, the findings indicate that they should be prioritized for further populational and laboratory studies. The nsSNPs in the *SCN9A* gene that have an impact in biological processes were studied using a method that combines the forecasts of various tools to identify the most genetic mutations. When the single sodium channel (Na1.7) is inactivated, all pain sensation is abolished (apart from nerve pain). Significant effects could be anticipated in the domain of analgesia if antagonist for Na1.7 might be identified. [79] The Na1.7 channel generally serves as gatekeeper channels, amplifying weak impulses and pushing neurons to threshold voltages where the far more complicated Na1.8 channels became activated. Because Na1.8 channels generate the majority of the current needed to produce action potentials and transduce pain, they are a key player in this pathway [80–83].

Moreover, the point mutation found in *SCN9A* enables the channels to be triggered by less significant depolarizing events and, as a result, have increased activity [84]. The *SCN9A* (NC 000002.12) gene encodes the Nav1.7 and spans 113.5 kb with 26 exons and mapped on human chromosome 2q24.3 [18,85]. Sodium channels produced by this gene and are mainly found in neurons of the DRG and the sympathetic ganglia. Each domain of this Na^+^ channel, which contains 1988 amino acid is split into six membrane segments. [18–20,29–31] Further, *SCN9A* polymorphisms have been linked to a spectrum of pain perception, from extreme insensitivity to intense hypersensitivity, by encoding the alpha monomer of NaV1.7 channels was attributed to variations in pain sensitivity [12–15]. A new nonsense mutant R523X, which affects the first linker that joins domains 1 and 2, was discovered in exon 10 of a Pakistani family. The same linker, S459X has previously been associated with loss of function, indicating that this site is essential for the development of the channel [18]. The patient had previously been found to be a homozygous carrier of the prevalent polymorphism in the *SCN9A* gene, which codes for the NaV1.7-dbSNP rs6746030 (R1150W). This SNP was originally believed to be part of quantitative changes in the pain threshold in various patient cohorts rather than being associated with erythromelalgia [86].

Likewise, A study found a significant association between pain intensity and SNP rs6746030. The Nav1.7 coding sequence is modified by the two alleles of the rs6746030 [18]. These were independently transduced into HEK293 cells, and patch-clamping was used to evaluate their electrophysiological effects. They concluded that pain perception varies in response to nociceptive stimuli based on the *SCN9A* rs6746030 genotype. The patient was found to be heterozygous for the SCN9A gene mutation c.4384T>A (p.F146I), which is related with PEPD [29]. Primary erythromelalgia (PEM) is an autosomal dominant disorder caused by *SCN9A* gene mutations, and these variants (Q10R, I136V, P187, S211T, F216S, I228M, I234T, S241T, N395K, V400M, P610T, G616R, DII, L823R, F826Y, I848T, G856R, L858H, L858F, A863P, Q875E, DelL955, R1150W, DIII, N1245S, DIV, W1538R, A1632E and A1632G) are strongly linked to PEM. In 2004, Yang et al [87,88] were the first to link the disorder to *SCN9A* gene.

The biophysical properties of Nav1.7 are altered in a similar manner by all PEM mutations; this alteration involves a shift of the activation voltage to hyperpolarized potentials, the magnitude of which appears to correlate with the severity of symptoms; [88] while the variants R996C, V1298D, V1298F, V1299F, I1461T, F1462V, T1464I, A1632E,M1627K,G1607R, I228M, R185H, L1612P and V1740L have reported *SCN9A* mutations that are found Paroxysmal extreme pain disorder (PEPD, formerly known as familial rectal pain syndrome) patients, is caused by gain of function mutations in *SCN9A* that alter the biophysical properties of the Nav1.7 channel [19,88–92]. People who had the PEM polymorphism N1245S showed greater olfactory sensitivity, whereas the variation N641Y, Q10R, G327E, I775M, R429C, and Y1958C experienced epilepsies and lost their smell sense due to the activation of Nav1.7 in the olfactory epithelia [93,94]. It was found that several mutations exist in 1 and 2 domain, the majority of which are nonsense variants that result in proteins being cut off prematurely [88]. Some polymorphisms such as S459X, I767X, W897X, R277X, Y328X, E693X, R830X, F1200L, R1488X, K1659X, I1235L, W1689X, R523X, R896Q, K370Q, G375A, E919X, M1190X, G1822, R896G AND Q369X) were identified as involved in complete insensitivity to pain [18,19,35,43,79,81,88,95].

Fig 2 shows the outcomes of a query of the NCBI and Uniprot databases for pathogenic nsSNPs. The polymorphisms defined as SCN9A-associated or pathogenic in the dbSNP database including E1889D, L1802P, F1782V, D1778N, C1370Y, V1311M, Y1248H, F1237L, M936V, I929T, V877E, D743Y, C710W, and D623H were predicted as harmful consequences in 14 tools, while 5 SNPs (L1802P, F1782V, D1778N, V1311M and M936V) were thoughts to be highly deleterious. Homology modeling with SWISS Model was used to generate a 3D model of the protein sequences of both wild-type (SCN9A) and mutants, allowing us to investigate the effects of these 5 variations. High RMSD mutants, including L1802P, F1782V, D1778N, V1311M, and M936V, are well-known to be incredibly detrimental. Thus, PyRx was brought to use for protein-ligand interaction. Using PyRx, we docked 20 ligands to both wild-type and mutated SCN9A (L1802P, F1782V, D1778N, V1311M, and M936V). The binding affinity of ligand-receptor compounds was used to evaluate the index value at each site. Docking was developed to study how ligand binding activity correlates with three-dimensional protein structure. Research method used in this research common underlying on creating an association between the modifications and their molecular consequences on the protein. Since each program/tool performs on a distinct algorithm, the results are more reliable when multiple are utilized to accomplish a single purpose.

## 4. Conclusions

Genetic analysis for identifying phenotypic or disease-associated polymorphism is a complex situation that demands for advanced techniques. We investigated a variety of methods to determine the variations in the human *SCN9A* gene that are likely be damaging. The voltage-gated sodium-channel type IX subunit Nav1.7, which is located in peripheral neurons and is encoded by the SCN9A gene, is vital to the generation of action potentials. The *SCN9A* mutations were associated with primary erythermalgia, channelopathy-associated insensitivity to pain, and paroxysmal intense pain condition. In this study, 14 nsSNPs with a mutational effect on the SCN9A function and structure were found to be highly deleterious, according to the trajectory analysis and stepwise prediction of pathogenicity of nsSNPs (SNPNexus > SNAP2 > PolyPhen 2> Mutpred2>PANTHER> CADD> ConDEL> P-Mutant> Meta SNP> PhD SNP> SNP & GO). Five nsSNPs have been examined as deleterious SNPs in the *SCN9A* protein using a structural homology-based method by the Swiss model. In the present study, we analyzed the L1802P, F1782V, D1778N, V1311M and M936V SNPs associated with the *SCN9A* gene was docked with selected 9 molecules with significant binding affinities including Batrachotoxin, Carbamazepine, Flecainide, Funapide, Naloxone, Phenytoin, Ranolazine, Saxitoxin and vixotrigine and docked with native and mutant structures and visualized by Discovery studio. Future genome association studies will be able to detect damaging SNPs linked to specific patients with pain according to the findings of this study. To characterize this data on SNPs, extensive clinical trial based research on a broad population are needed, as well as experimental mutational studies for the confirmation of the outcomes.

## Abbreviations

GWAS: genome-wide association studies
nsSNPs: non-synonymous SNPs
VGSCs: voltage gated sodium channels
DRG: dorsal root ganglion
COMT: catechol-O-methyltransferase
SIFT: sorting intolerant from tolerant
LGA: lamarckian genetic algorithm
