# Correction: Identification and *In-Silico* study of non-synonymous functional SNPs in the human *SCN9A* gene

**DOI:** 10.1371/journal.pone.0301489

**Published:** 2024-03-26

**Authors:** Sana Waheed, Kainat Ramzan, Sibtain Ahmad, Muhammad Saleem Khan, Muhammad Wajid, Hayat Ullah, Ali Umar, Rashid Iqbal, Riaz Ullah, Ahmed Bari

In the Funding statement, the grant number from the funder King Saud University, Riyadh, Saudi Arabia, is listed incorrectly. The correct grant number is: RSP2024R110.

The affiliation for the eighth author is incorrect. The correct affiliation is not indicated. Rashid Iqbal is not affiliated with #5 but with the Department of Agroecology-Climate and Water, Aarhus University, Blichers Alle 20, 8830 Tjele, Denmark.
