# Expression of Concern: Identification and *In-Silico* study of non-synonymous functional SNPs in the human *SCN9A* gene

**DOI:** 10.1371/journal.pone.0345384

**Published:** 2026-03-20

**Authors:** The PLOS One Editors

The *PLOS One* Editors issue an Expression of Concern to alert readers that this article [1,2] was identified as one of a series of submissions for which we have concerns about adherence to the journal’s authorship policy. We regret that these issues were not addressed prior to the article’s publication.
